# Draft Genome of the Multidrug-Resistant Acinetobacter baumannii Strain A155 Clinical Isolate

**DOI:** 10.1128/genomeA.00212-15

**Published:** 2015-03-26

**Authors:** Brock A. Arivett, Steven E. Fiester, David C. Ream, Daniela Centrón, Maria S. Ramírez, Marcelo E. Tolmasky, Luis A. Actis

**Affiliations:** a Department of Microbiology, Miami University, Oxford, Ohio, USA; b Instituto de Microbiología y Parasitología Médica, Universidad de Buenos Aires, Consejo Nacional de Investigaciones Científicas y Tecnológicas, Buenos Aires (IMPaM, UBA-CONICET), Ciudad Autónoma de Buenos Aires, Argentina; c Department of Biological Science, Center for Applied Biotechnology Studies, California State University Fullerton, Fullerton, California, USA

## Abstract

Acinetobacter baumannii is a bacterial pathogen with serious implications on human health, due to increasing reports of multidrug-resistant strains isolated from patients. Total DNA from the multidrug-resistant A. baumannii strain A155 clinical isolate was sequenced to greater than 65× coverage, providing high-quality contig assemblies.

## GENOME ANNOUNCEMENT

Acinetobacter baumannii, a Gram-negative pathogen, causes a variety of nosocomial infections such as bacteremia, meningitis, skin and soft tissue infections such as necrotizing fasciitis, ventilator-associated pneumonia, and urinary tract infections (1, 2). These infections are becoming harder to treat due to the rise in the number of multidrug-resistant A. baumannii strains present in clinical settings (1, 3). A. baumannii strain A155 is a multidrug-resistant strain originally isolated from a urinary sample at a hospital in Buenos Aires, Argentina, in 1994 (4). At that time, unlike in most of the world where clonal complex 109 (CC109) and CC92 (also known as international clonal lineage 1 and 2, respectively) were predominant, most strains isolated in Argentina belonged to CC113 (5). A. baumannii A155 was among the first CC109 isolates in Argentina (4). This strain includes the AbaR-type island inserted within *comM*, and the *aac(6′)-Ib* gene, which confers resistance to numerous aminoglycosides (4, 6, 7).

A. baumannii A155 whole-genome sequencing and annotation were performed as described previously (8). Briefly, the A155 isolate was routinely stored at −80°C in 10% glycerol, passaged to overnight LB cultures grown with agitation at 37°C, and total DNA was isolated using the DNeasy blood and tissue kit (Qiagen, Valencia, CA, USA). DNA quantity and quality were assessed using Nanodrop 2000 (Thermo Scientific, Wilmington, DE, USA). The SYBR Green (Life Technologies, Grand Island, NY, USA) standard curve method was used to estimate DNA concentration for library preparation in a black 96-well plate (Corning, Tewksbury, MA, USA), and fluorescence values were obtained using a FilterMax F5 spectrophotometer with Multi-Mode Analysis software version 3.4.0.25 (Molecular Devices, Sunnyvale, CA, USA). The Nextera XT kit (Illumina, Inc., San Diego, CA, USA) was used to simultaneously fragment and construct adapter-tagged libraries per the manufacturer’s instructions. The Bioanalyzer 2100 High Sensitivity DNA analysis kit (Agilent Technologies, Santa Clara, CA, USA) with version B.02.08.SI648 software was used to determine the fragmentation of the resultant libraries. Individual libraries were normalized by bead-based affinity, pooled, and then sequenced using the MiSeq version 3 600-cycle kit (Illumina) to perform 300-bp paired-end sequencing on a MiSeq instrument (Illumina) per the manufacturer’s instructions. *De novo* assembly was performed using Genomics Workbench version 7.5 with the Bacterial Genome Finishing module (CLC bio, Boston, MA, USA) on a workstation with an AMD Opteron 2.10-GHz 16-core processor and 128-GB DDR3 ECC RAM. Genomes were annotated with Prokka version 1.10 on a quadcore i7 workstation with 32-GB DDR3 running Ubuntu 14.04 LTS (9).

The *de novo* assembly resulted in a 3,933,455-bp genome encoding 55 tRNAs and 3,760 genes with 3,704 proposed CDSs for the A. baumannii A155 clinical strain.

### Nucleotide sequence accession numbers.

The first version of the *de novo* whole-genome assembly of A. baumannii A155 was deposited into GenBank under Bioproject ID PRJNA261239 with the accession number JXSV00000000, version JXSV01000000.
